# Supplementary material for: Daily Yogurt Consumption Improves Glucose Metabolism and Insulin Sensitivity in Young Nondiabetic Japanese Subjects with Type-2 Diabetes Risk Alleles
Source: Nutrients. 2018 Nov 29;10(12):1834. doi: 10.3390/nu10121834 (PMC6316314; doi:10.3390/nu10121834)
Supplement: Supplementary file 1 [file nutrients-10-01834-s001.pdf]

Supplemental Table 1. Primers and probes used for real-time PCR (HybProbe assays).

Locus	Type	Primer and probe sequences
KCNQ1 (rs2237892)	Forward primer	TGCATCCTAAGGTGGTTCAG (20)
	Reverse primer	TGGTAGGGAACAACTGGAGAC (21)
	Probe 1	LC Red 640-TCCTGTGACAGCTCCCATCATCTGGC-Phosphate (26)
	Probe 2	ACCCC**A**GGTGGCAAA-Fluorescein (15)
CDKAL1 (rs2206734)	Forward primer	CAAACAACAGCAGGGACTTA (20)
	Reverse primer	AACTCAGCATGGGTAATAGGTAG (23)
	Probe 1	LC Red 640-ATAC**C**GTAAACACCAGAATG-Phosphate (20)
	Probe 2	GAAAGTGATGAGAGATAGGATGACCACATA-Fluorescein (30)
CDKN2B (rs2383208)	Forward primer	ACTGTACTATCTAGTAGCTAATCTGTC (27)
	Reverse primer	CTGCACCACTGGAGGTAA (18)
	Probe 1	LC Red 640-CCTTCCTGTCACAGTTTTAGTTGCTAATCACATAA CATTAAGAAACC-Phosphate (47)
	Probe 2	CTCAATTCATG**C**GGACTT-Fluorescein (18)
UBE2E2 (rs6780569)	Forward primer	TCAGGTCATGTCCTTGCAG (19)
	Reverse primer	GCTCAGGGTCTTTTCAACAC (20)
	Probe 1	ATGATTTTGACATTGGCAGGGTGATAAAAGGGAGA-Fluorescein (35)
	Probe 2	LC Red 640-TTGAGAGTAT**A**GAGGGAAGA-Phosphate (20)
IGF2BP2 (rs1470579)	Forward primer	AAATGGCTACTGCAACTAAGAC (22)
	Reverse primer	CTGCCACATGAAAATCTGTATC (22)
	Probe 1	TTTGAGTTTCCAAACAGCTATCATCATTAGATAAGATCC-Fluorescein (39)
	Probe 2	LC Red 640-TACGAGTT**C**ATCCTGCC-Phosphate (17)

Target genes are shown in locus. Number in parentheses indicates the length of the primers and probes. Listed after primer sequences are the detection probe and then the anchor probe. Nucleotides in bold in the detection probe indicate the locations of the SNPs. CDKAL1; CDK5 regulatory subunit associated protein 1-like 1, CDKN2B; cyclin-dependent kinase inhibitor 2B, IGF2BP2; insulin-like growth factor 2 mRNA binding protein 2, KCNQ1; potassium voltage-gated channel, KQT-like subfamily, member 1, LC Red 640, LightCycler-Red 640-N-hydroxy-succinimide ester; PCR, polymerase chain reaction; SNP, single nucleotide polymorphism, UBE2E2; ubiquitin-conjugating enzyme E2.

Supplemental Table 2. Effect of habitual yogurt consumption on stool frequency, stool consistency, feeling of incomplete evacuation and difficulty evacuation, and abdominal bloating.

Parameters	Before intervention	After intervention				*p* Value [a]
		1 week	2 weeks	3 weeks	4 weeks	
Stool frequency (times/day)	1.0 ± 0.6	1.0 ± 0.6	1.1 ± 0.6	1.2 ± 0.6	1.4 ± 0.7 *	**0.038**
Stool frequency (days/week)	5.3 ± 1.6	5.4 ± 1.6	5.5 ± 1.3	5.8 ± 1.0	6.1 ± 0.8 *	**0.042**
Stool consistency (BSS) [b]	2.9 ± 0.5	2.9 ± 0.6	2.8 ± 0.7	3.0 ± 0.7	3.0 ± 0.6	0.595
Feeling of incomplete evacuation [c]	0.7 ± 0.7	0.5 ± 0.6	0.6 ± 0.8	0.5 ± 0.6	0.5 ± 0.5	0.505
Feeling of difficulty evacuation [c]	0.5 ± 0.6	0.7 ± 0.6	0.6 ± 0.7	0.5 ± 0.6	0.5 ± 0.6	0.761
Abdominal bloating [d]	0.7 ± 0.8	0.7 ± 0.7	0.7 ± 0.7	0.6 ± 0.5	0.5 ± 0.5	0.667

Values are shown as mean ± standard deviation (SD). BSS, Bristol Stool Scale. [a] A multiple-comparison test (Dunnett-Hsu post-hoc analysis) was used to compare the differences in means between before and after intervention (compared with before intervention). Bold values and "*" sign are statistically significant ($p < 0.05$). [b] The four types of stool are;1 = separate hard lumps, like nuts (hard to pass) or watery, no solid pieces, entirely liquid; 2 = sausage-shaped, but lumpy or fluffy pieces with ragged edges, mushy stool; 3 = like a sausage but with cracks on its surface or soft blobs with clear cut edges (passed easily); 4 = like a sausage or snake, smooth and soft. [c] The four types of feeling are; 0 = no distention; 1 = not very severe; 2 = quite severe; 3 = severe, 4 = very severe. [d] The four types of feeling are; 0 = no feeling; 1 = not very feeling; 2 = quite feeling; 3 = feeling, 4 = very feeling.
